# Metal Oxide Hydrogel Composites for Remediation of Dye-Contaminated Wastewater: Principal Component Analysis

**DOI:** 10.3390/gels8110702

**Published:** 2022-10-30

**Authors:** Nimer Murshid, Omar Mouhtady, Mahmoud Abu-samha, Emil Obeid, Yahya Kharboutly, Hamdi Chaouk, Jalal Halwani, Khaled Younes

**Affiliations:** 1 College of Engineering and Technology, American University of the Middle East, Kuwait; 2 Water and Environment Sciences Lab, Lebanese University, Tripoli, Lebanon

**Keywords:** dye removal, hydrogel, hydrogel composites, machine learning, metal oxide nanoparticles, principal component analysis, wastewater

## Abstract

Water pollution is caused by multiple factors, such as industrial dye wastewater. Dye-contaminated water can be treated using hydrogels as adsorbent materials. Recently, composite hydrogels containing metal oxide nanoparticles (MONPs) have been used extensively in wastewater remediation. In this study, we use a statistical and artificial intelligence method, based on principal component analysis (PCA) with different applied parameters, to evaluate the adsorption efficiency of 27 different MONP composite hydrogels for wastewater dye treatment. PCA showed that the hydrogel composites CTS@Fe_3_O_4_, PAAm/TiO_2_, and PEGDMA-rGO/Fe_3_O_4_@cellulose should be used in situations involving high pH, time to reach equilibrium, and adsorption capacity. However, as the composites PAAm-co-AAc/TiO_2_, PVPA/Fe_3_O_4_@SiO_2_, PMOA/ATP/Fe_3_O_4_, and PVPA/Fe_3_O_4_@SiO_2_, are preferred when all physical and chemical properties investigated have low magnitudes. To conclude, PCA is a strong method for highlighting the essential factors affecting hydrogel composite selection for dye-contaminated water treatment.

## 1. Introduction

Dyes are made of synthetic organic material. They are mutagenic and exhibit biological toxicity, such as teratogenicity and carcinogenicity [1,2]. Dyes are primarily used in the production of some consumer goods, including textiles, plastics, paints, paper, and printing inks. According to recent studies [3], approximately 60,000 tons of dyes are discharged annually worldwide. Synthetic and organic dyes are mainly produced through the textile dyeing process. Azo dyes, which correspond to more than half of the total global production of dyes, represent a major part of artificial dyes [4,5]. Due to their complex molecular structure, synthetic dyes are known to be refractory to temperature [6], and very stable; hence, they are not easily biodegradable [7]. Subsequently, dye-contaminated water discharged by industries is one of the major water pollution issues threatening drinking water supplies [8].

Huge efforts and numerous physical, chemical, and biological remediation methods have been devoted to the treatment of the aquatic environment [9]. In particular, physical processes, including adsorption, show promising and long-term sustainable efficacy in treating dye-contaminated water [10]. Indeed, adsorbent materials are very capable of eliminating contaminants [11]. By definition, adsorption is a surface phenomenon in which a solute adheres to a solid sorbent. The solute can be an atom, ion, or molecule in a gas or liquid state. Adsorption processes have several advantages over other methods, such as filtration, precipitation, coagulation, reverse osmosis, ion exchange, and oxidative processes. In addition, adsorption processes are effective against a wide range of pollutants while keeping a simple design and a low cost [12,13,14]. When dye-contaminated waters, hydrophilic materials, and functional materials are considered, there is a tendency to favor better improved adsorption results. Recently, the use of composite hydrogels for adsorption has been the focus, thanks to their promising properties in comparison to conventional hydrogels or some other hydrophilic materials [10]. Due to their three-dimensional network structure and polymeric hydrophilicity, hydrogels are able to adsorb large quantities of water and to swell while preserving their structures. This is due to individual polymer chains that are chemically or physically cross-linked [10]. These composites can also be enriched with a variety of functional groups to further improve the adsorption of dyes and heavy metal ions from aqueous media.

Recently, composite hydrogels containing metal oxide nanoparticles (MONPs) have been extensively prepared as they have been used in different areas, including environmental remediation [15,16,17]. The use of these composites in the treatment of dye-contaminated water has received particular attention [10,18,19]. MONPs have numerous characteristics, such as specific adsorption properties [20], magnetic features, and redox capabilities. Therefore, in addition to their ability to improve hydrogels’ electrical, mechanical, and thermal properties, MONPs have been used to enhance adsorption selectivity and catalytic activity in pollutant species degradation [21,22]. By adjusting the external magnetic field, they can also allow for remote control of swelling and analyte adsorption/desorption. In fact, composite hydrogels containing magnetic MONPs can reversibly change shape and volume in response to external magnetic fields [23,24,25]. Because an external magnetic field imposes attractive/repulsive forces, the movements of the embedded magnetic nanoparticles direct the polymeric chains’ contraction and distention [26]. As a result, liquid diffusion throughout the hydrogel matrix can be tailored, influencing the adsorption/desorption of the concerned solutes, such as dye molecules. The ability to easily recover from the treated media using magnets represents one more advantage for composite hydrogels containing magnetic compounds when compared to the use of more arduous processes, such as filtration, sedimentation, or centrifugation [27,28].

Principal component analysis (PCA) with several parameters was used to assess the adsorption efficiency of composite hydrogels containing MONPs in wastewater dye removal. PCA is generally used to reduce the parameters of a dataset by generating linear combinations of the original parameters, and thus to identify the main parameters required to enhance and improve a given process [29,30,31]. Following the huge number of parameters affecting the effectiveness of composite hydrogels containing MONPs for wastewater treatment, a PCA study can be implemented to pursue intercorrelation in parameters associated with adsorption efficiency. In this work, we used the same methodology as our previously published work on dye removal using graphene oxide hydrogels [29]. Herein, we conduct our analysis on 27 different MONPs hydrogel composites, and we examine the intercorrelation between five parameters, namely pH, adsorbent dosage (D), time to reach equilibrium (ET), adsorption surface (qm), and the content of MONPs in the hydrogel (MONP%). To the best of our knowledge, this is the first statistical and artificial intelligence study that has been used to assess the adsorption efficiency of MONPs containing hydrogels for dye removal.

## 2. Results and Discussion

**Figure 1:**
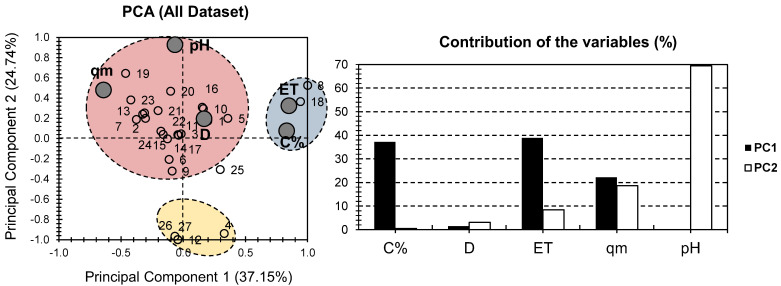
PCA for all datasets. Small empty bullets represent the 27 investigated hydrogels containing MONPs. Large gray bullets represent different physical and chemical properties.

PCA analysis was conducted on previously published data (Table 1) from the study of Pereira et al. [10]. Figure 1 presents the PCA bi-plot for previously published data on the physical and chemical properties of various composite hydrogels containing MONPs used for dye removal from water [10]. The first two PCs were responsible for 61.89% of the total variance (37.15% for PC1 and 24.74% for PC2) (Figure 1). When the physical and chemical properties of composite hydrogels containing MONPs (and derivatives) were considered, they yielded similar results to those of PCA [29]. This indicates that the PCA approach is equally efficient for both dataset approaches. ET provided the highest contribution to PC1 for the factor MONP% and accounted for 76% of its total contribution. The high contribution of MONP% was surprising, given that fewer data for this factor were provided following the various investigated samples (Table 1). In terms of PC2, pH was the most significant factor, accounting for 70% of its total contribution. The large disparity in factor contribution following the first two PCs indicates the representability of the investigated physical and chemical properties for the various hydrogels under consideration. MONP% and ET had a strong positive influence along PC1, with no to minor positive influence along PC2. This could probably indicate a high correlation between the necessary time to reach equilibrium, from one side, and the carbon content, from the other side. Nonetheless, this could not be confirmed or infirmed following the shortage in data with regard to the carbon content. For pH, it showed a high positive influence along PC2, with no influence along PC1. As for qm, it showed an average negative influence, and a positive influence along PC1, and PC2. Interestingly, D showed nearly no influence on either PC.

Individuals can be clustered in three ways (blue, red, and yellow) based on the different trends found in the samples (Figure 1). Surprisingly, the red cluster contained the vast majority of the samples examined. This cluster, along with D, qm, and pH, was positively correlated, indicating that these properties had the greatest influence on the investigated hydrogels. It put together samples 4, 12, 26, and 27 for the yellow cluster. All investigated factors had a negative to low correlation with these samples. This suggests that these hydrogels could be used in situations where low pH, adsorbent dosage (D), time to reach equilibrium (ET), and adsorption surface (qm) are required. Only samples 8 and 18 were collected for the blue cluster because they were positively correlated with ET and MONP%. Interestingly, both hydrogels included ferric oxide in their composite structure, despite the fact that this feature is not unique to them. In summary, when the entire dataset was considered, the PCA presentation demonstrated an acceptable presentation of the truth (around 60% of the total variance; Figure 1). However, one shortcoming may arise from the fact that MONP% was missing for the majority of the investigated hydrogels. This will almost certainly create a bias in the differences. As a result, overcoming this problem is as simple as ignoring the MONP% portion.

**Figure 2:**
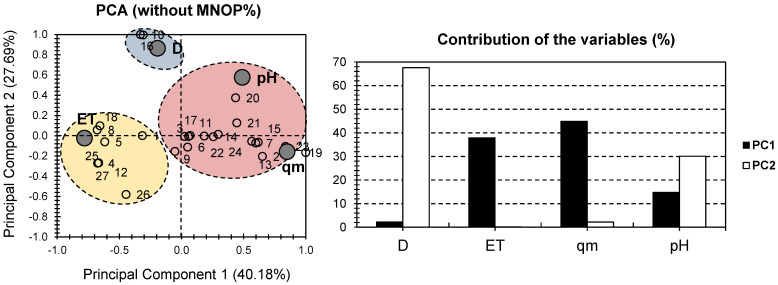
PCA for all datasets. Small empty bullets represent the 27 investigated hydrogels containing MONPs. Large gray bullets represent different physical and chemical properties, with the exclusion of MONP%.

The PCA bi-plot for the physical and chemical properties of the investigated hydrogels, excluding MONP%, is shown in Figure 2. The first two PCs were responsible for 67.87% of the total variance (40.18% for PC1 and 27.69% for PC2; Figure 2). The higher variance score, in comparison to the PCA bi-plot in Figure 1, indicates that the strategy used was effective. ET and qm were the factors that contributed the most to PC1, accounting for 82.85% of the total contributions. In terms of PC2, D contributed the most (67.62%), with pH having a moderate influence (30% of the PC2 contribution; Figure 2). Similar to the case in Figure 1, a high discrepancy in the factors’ contributions is scored. Figure 2 shows a higher distribution of the factors, which is interesting. On one side of PC1, qm had a strong positive influence, while ET had a strong negative influence. D had a significant positive influence on PC2. Both positive influences on pH were observed in both PCs.

Individually, and similarly to the “all dataset” case, three distinct clusters can be identified when MONP% is considered (Figure 2). The majority of the samples were found in the red cluster, which is positively correlated with both pH and qm. The yellow cluster contained a smaller number of samples than the red cluster. It included samples 1, 4, 5, 8, 12, 18, 25, and 27, all of which showed a strong positive correlation with ET. Interestingly, more samples were more likely to be influenced by the time to reach equilibrium when MONP% was excluded. Only hydrogel samples 10 and 16 were found in the blue cluster, which was positively correlated with D. Nonetheless, the lack of data input for these two samples makes a non-speculative conclusion about the origin of this proximity impossible. In summary, when the MONP% was excluded, the dataset’s representativeness increased. This is demonstrated by the greater contribution of total variance in Figure 2 than in Figure 1. A “separation of individuals” approach was used to improve the presentation of the dataset. The goal was to perform a PCA on each cluster to gain a better understanding of the similarities and differences between the hydrogel samples under consideration.

**Figure 3:**
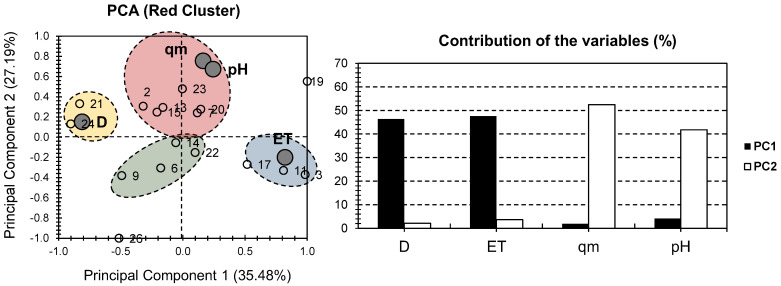
PCA for all datasets. Small bullets represent the 15 investigated hydrogels containing MONPs (red cluster components of Figure 2). Large gray bullets represent different physical and chemical properties, with the exclusion of metallic oxide nanoparticles (MONP%).

Figure 3 presents the PCA bi-plot for the physical and chemical properties of the samples of the red cluster in Figure 2. The first two PCs accounted for 62.58% of the total variance (35.48% for PC1, and 27.19% for PC2; Figure 3). For the factors, the highest contribution was scored for D and ET, along PC1 (46.38% and 47.57%, respectively). For PC2, the highest contribution was scored for qm and pH (52.47% and 41.74%, respectively). Interestingly, a high distribution of the factors can be noticed, as in the case of Figure 2. ET had a strong positive influence on one side of PC1, while D had a strong negative influence on the other. Both pH and qm had a significant positive influence along PC2, with a minor positive influence along PC1.

For individuals, four different clusters were distinguished. The red cluster contained samples 2, 7, 13, 15, 20, and 23 and showed a positive correlation along qm and pH factors. The blue cluster contained samples 3, 11, and 17 and showed a positive correlation along the ET. The yellow cluster only contained samples 21 and 24 and showed a positive correlation with factor D. As for the green cluster, it contained samples 6, 9, 14, and 22 and showed a negative correlation along all of the investigated factors. Even though the red cluster PCA presented a lower variance than the “all dataset” approach, it similarly showed a higher distribution of the factors along the first two PCs, and it distinctively showed a high distribution of the individuals (four clusters in Figure 3, rather than three clusters in Figure 2). This allows for a better distinction between the different features and conditions of the different investigated hydrogel composites.

**Figure 4:**
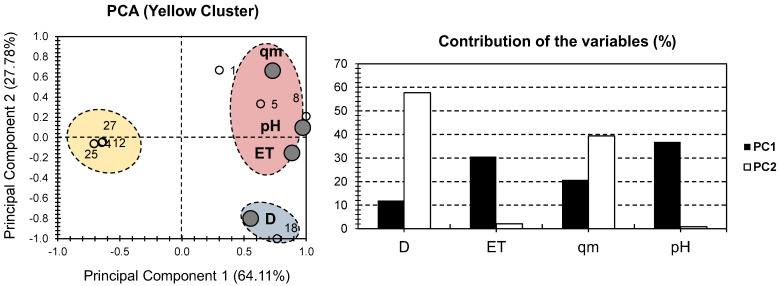
PCA for all datasets. Small bullets represent the 15 investigated hydrogels containing MONPs (yellow cluster components of Figure 2). Large gray bullets represent different physical and chemical properties, with the exclusion of metallic oxide nanoparticles (MONP%).

Figure 4 depicts the PCA bi-plot for the physical and chemical properties of the samples in the yellow cluster of Figure 2. When only these samples were considered, a higher presentation of the total variance was observed, with a variance of 91.89% (64.11% for PC1 and 27.78% for PC2; Figure 4). This demonstrates the effectiveness of the strategy used, as more focus on “similar” individuals’ results in a greater ability to compare them. In the case of PC1, the factors with the highest contributions were pH and ET (36.8% and 30.56%, respectively). In terms of PC2, D had the highest contribution (57.72 percent of the total contribution of PC1), while qm had a moderate contribution of 40% (Figure 4). When compared to the original PCA in Figure 2, a lower distribution of the factors were seen along the bi-plot of the yellow cluster. As a result, all of the factors were located on the positive side of PC1, with qm on the positive side of PC2 and D on the negative side. It had no effect on pH or ET along PC2.

Individuals were divided into three clusters; the red cluster contained samples 1, 5, and 8, and had a high positive correlation with qm, pH, and ET. The blue cluster contained only hydrogel sample 18 and demonstrated a strong positive correlation with D. The yellow cluster contained samples 4, 12, 25, and 27, and it was located on the opposite side of the different factors (along the negative side of PC2). Given the high variance, it is safe to assume that the composite hydrogel CMSt/PVA/Fe_3_O_4_ should be used with high adsorbent doses. For CTS@Fe_3_O_4_, PAAm/TiO_2_, and PEGDMA-rGO/Fe_3_O_4_@cellulose, it should be used where high pH, time to reach equilibrium, and adsorption capacity were implemented. For PAAm-co-AAc/TiO_2_, PVPA/Fe_3_O_4_@SiO_2_, PMOA/ATP/Fe_3_O_4_, and PVPA/Fe_3_O_4_@SiO_2_, it should be used where all of the investigated factors are low.

## 3. Conclusions

In this study, we performed principal component analysis (PCA) for a better understanding of the correlation between several chemical and physical properties. The properties in-hand are: (a) Time to reach equilibrium (ET), (b) water acidity (pH), (c) Adsorbent dosage (D), and (d) adsorption capacity (qm). In order to seek a higher presentation of the dataset, a “separation of individuals” approach was acquired. The aim was to perform a PCA on each of the clusters to better seek the similarities and dissimilarities of the investigated hydrogel composites. Interestingly, a higher presentation of the total variance was shown in one of the cases, making the PCA-biplot reliable for seeking solid conclusions. The PCA (Figure 4) showed different potential applications for some of the investigated hydrogels. In fact, CTS@Fe_3_O_4_, PAAm/TiO_2_, and PEGDMA-rGO/Fe_3_O_4_@cellulose should be used where high pH, time to reach equilibrium, and adsorption capacity are encountered. For PAAm-co-AAc/TiO_2_, PVPA/Fe_3_O_4_@SiO_2_, PMOA/ATP/Fe_3_O_4_, and PVPA/Fe_3_O_4_@SiO_2_, it should be used where all of the investigated physical and chemical properties are at low magnitudes. A shortcoming arising from this study resides in the neglecting of structural differences between azo dyes compounds. In fact, we aimed to focus on the physical and chemical features of the adsorbent. Therefore, we have assumed that all azo dyes compounds have similar adsorption properties. Hence, it could be interesting to investigate the influence of adsorbent molecular discrepancies in further investigations.

## 4. Methodology

The purpose of this study is to apply PCA to a previously published study by Pereira et al. [10] (Table 1) in order to better understand the differences in the functioning of multiple metal oxide nanoparticle (MONP)-based hydrogels based on their adsorption properties. PCA is regarded as a technique for identifying patterns among variables. The bi-dimensional statistical approach failed to reveal these patterns. It presents an unsupervised machine-learning method because, once applied, no prior knowledge of the data or the investigated phenomena is assumed. A unit-weighting vector (*W_j_*) and the original data matrix *M* with m × n dimensions (m: number of variables, n: number of datasets) are used to express the *j*th PC matrix (*Pj*) [31,54,55].

(1) $$Pj=WM=\sum_{}^{i=0}W_{ji}M_{i}$$

where *W* is the loading coefficient and *M* is the n-dimensional data vector. *M*(*Var*(*M*)), which is obtained by projecting *M* to *W*, should be maximized as follows:

(2) $$Var\left(M\right)=\frac{1}{n}\;\left(W^{T}M\right){\left(WM\right)}^{T}=\frac{1}{n}\;W^{T}MM^{T}W$$

(3) $$MaxVar\left(M\right)=Max\left(\left(\frac{1}{n}\right)\;W^{T}MM^{T}W\right)\;$$

Since $\frac{1}{n}\;MM^{T}$ is the same as the covariance matrix of *M*(*cov*(*M*)), *Var*(*M*) can be expressed as follows:

(4) $$Var\;\left(M\right)=W^{T}cov\;\left(M\right)\;W$$

The Lagrangian function can be defined using the Lagrange multiplier method, which is as follows:

(5) $$L=W^{T}$$

(6) $$L=W^{T}cov\left(M\right)W-\delta \left(W^{T}W-1\right)\;$$

Because the weighting vector is a unit vector, “*W^T^W* − 1” is assumed to be equal to zero in Equation (6). As a result, the maximum value of *Var*(*M*) can be calculated by equating the derivative of the Lagrangian function (*L*) with respect to *W,* as follows:

(7) $$\frac{dL}{dW}=0$$

(8) cov(M)W−δW=(cov(M)−δI)W=0 

where, *δ*: eigenvalue of *cov*(*M*), *W*: eigenvector of *cov*(*M*).

## Figures and Tables

**Table 1:** Physical and chemical properties data of different composite hydrogels containing MONPs used for the removal of dyes from water (adapted with permission from Pereira et al. Ref [10]).

MONPs Composite Hydrogel	Composite #	MONP%	D	ET	qm	pH	Ref
CTS@ Fe_3_O_4_	1	-	1	400	142	7	[32]
ALG@Yttrium	2	-	2	30	1087	6	[33]
Collagen-*g*-PAAc-*co*-NVP/Fe_3_O_4_@SiO_2_	3	-	0.05	150	199	7	[34]
PAAm-*co*-AAc/TiO_2_	4	20	1	-	2.2	-	[35]
PAAm/TiO_2_	5	0.5	-	600	132	6.5	[36]
St-*g*-PAAc/ZnSe	6	-	1	30	189	6	[37]
PAAc/Co_3_O_4_	7	-	0.5	30	837	-	[38]
PEGDMA-rGO/Fe_3_O_4_@cellulose	8	30	2.5	720	112	7.4	[39]
CTS/Fe_3_O_4_@*κ*-CARR	9	-	2	30	123	5.5	[40]
CTS/MMT/γFe_2_O_3_	10	-	100	180	82	-	[41]
Collagen-*g*-PAAc-*co*-NVP/Fe_3_O_4_@SiO_2_	11	-	0.05	125	202	7	[34]
PVPA/Fe_3_O_4_@SiO_2_	12	0	1.4	-	14	-	[42]
AMPS/NIPAAm/Fe_3_O_4_	13	0	1	10	833	7	[43]
AMPS/NIPAAm/Cu_2_O	14	0	1	35	341	7	[43]
AMPS/NIPAM/Fe_3_O_4_·Cu_2_O	15	0	1	5	746	7	[43]
Cellulose/*κ*-CARR/TiO_2_	16	0.7			115	7	[44]
ALG/AgNPs	17		1	120	214	-	[45]
CMSt/PVA/Fe_3_O_4_	18		10	600	24	7	[46]
PAAm/CTS/Fe_3_O_4_	19		0.1	125	1603	7	[47]
Cellulose/Fe_3_O_4_-diatomite	20		0.7	30	102	10	[48]
HPG@Fe_3_O_4_	21		4	30	459	8	[49]
PAAc-*co*-AAm/Co_3_O_4_·Cu_2_O	22		0.5	40	238	7	[50]
PAAc-*g*-ALG/TiO_2_	23		0.6		1157	7	[51]
HPG@Fe_3_O_4_	24		4	30	400	7	[49]
PMOA/ATP/Fe_3_O_4_	25	3		400	1.7	4.6	[52]
PAAc-*g*-salep/AgNPs	26		1	20	93	2	[53]
PVPA/Fe_3_O_4_@SiO_2_	27		1.4		16	-	[42]

MONP% = Content of metal oxide (and derivatives) (wt-%) in the composite hydrogel. D = Adsorbent dosage (g/L). ET = time necessary to achieve the equilibrium condition (min). qm = Adsorption capacity (mg/g).

## Data Availability

The manuscript has no associated data.
